# High-throughput and separating-free phenotyping method for on-panicle rice grains based on deep learning

**DOI:** 10.3389/fpls.2023.1219584

**Published:** 2023-09-18

**Authors:** Yuwei Lu, Jinhu Wang, Ling Fu, Lejun Yu, Qian Liu

**Affiliations:** ^1^ Key Laboratory of Biomedical Engineering of Hainan Province, School of Biomedical Engineering, Hainan University, Haikou, China; ^2^ Britton Chance Center for Biomedical Photonics, Wuhan National Laboratory for Optoelectronics, Huazhong University of Science and Technology, Wuhan, Hubei, China; ^3^ MoE Key Laboratory for Biomedical Photonics, Huazhong University of Science and Technology, Wuhan, Hubei, China; ^4^ Department of Physics, School of Science, Hainan University, Haikou, China

**Keywords:** rice, rice panicle traits, high-throughput phenotyping, visible light scanning, deep learning

## Abstract

Rice is a vital food crop that feeds most of the global population. Cultivating high-yielding and superior-quality rice varieties has always been a critical research direction. Rice grain-related traits can be used as crucial phenotypic evidence to assess yield potential and quality. However, the analysis of rice grain traits is still mainly based on manual counting or various seed evaluation devices, which incur high costs in time and money. This study proposed a high-precision phenotyping method for rice panicles based on visible light scanning imaging and deep learning technology, which can achieve high-throughput extraction of critical traits of rice panicles without separating and threshing rice panicles. The imaging of rice panicles was realized through visible light scanning. The grains were detected and segmented using the Faster R-CNN-based model, and an improved Pix2Pix model cascaded with it was used to compensate for the information loss caused by the natural occlusion between the rice grains. An image processing pipeline was designed to calculate fifteen phenotypic traits of the on-panicle rice grains. Eight varieties of rice were used to verify the reliability of this method. The R^2^ values between the extraction by the method and manual measurements of the grain number, grain length, grain width, grain length/width ratio and grain perimeter were 0.99, 0.96, 0.83, 0.90 and 0.84, respectively. Their mean absolute percentage error (MAPE) values were 1.65%, 7.15%, 5.76%, 9.13% and 6.51%. The average imaging time of each rice panicle was about 60 seconds, and the total time of data processing and phenotyping traits extraction was less than 10 seconds. By randomly selecting one thousand grains from each of the eight varieties and analyzing traits, it was found that there were certain differences between varieties in the number distribution of thousand-grain length, thousand-grain width, and thousand-grain length/width ratio. The results show that this method is suitable for high-throughput, non-destructive, and high-precision extraction of on-panicle grains traits without separating. Low cost and robust performance make it easy to popularize. The research results will provide new ideas and methods for extracting panicle traits of rice and other crops.

## 1 Introduction

Rice (*Oryza sativa*) is one of the most important food crops in the world. Ensuring its yield and quality is crucial for food security and social and economic stability in the world (Tester and Langridge, 2010; Yang et al., 2014). At the same time, with the continuous development of the world’s socio-economic situation, people’s demand for food quality will not decline. Cultivating high-quality rice varieties with high yields has always been an important research direction of rice breeding (Zhang, 2007). The grain trait of rice is one of the most basic and essential rice breeding indexes, which directly reflects the grain yield and quality, including the total grain number, grain length, grain width, length-width ratio, the 1000-grain weight of a rice plant or a panicle and other traits (Li et al., 2019). However, unlike the highly developed genomic tools, the current phenotyping method of rice panicle and grain traits mainly relies on manual counting, which limits the efficiency and accuracy of panicle and grain trait statistics (Crossa et al., 2017; Watt et al., 2020; Sun et al., 2022).

Over the past few decades, researchers commonly obtained traits such as rice grain number and size by threshing and manually measuring (Crossa et al., 2017). This method is inefficient and straightforward to introduce the subjective error of the operator, and the destructive threshing operation will also affect the accuracy of the results (Huang et al., 2013). With the rapid development of computer vision and machine learning technology, automated and high-throughput crop phenotypic techniques based on various imaging techniques and image processing algorithms are gradually becoming essential for obtaining key phenotypic traits (Yang et al., 2020). Many researchers have made a series of beneficial explorations in automatically extracting rice grain-related traits.

The current research on extracting rice panicle-related traits can be divided into two categories from the perspective of pretreatment methods: requiring threshing and not requiring threshing. The method that requires threshing involves the destructive processing of rice panicles with the help of specialized threshing and conveying equipment, ultimately flattening the grains on an imaging platform and automatically calculating the quantity and size characteristics of the grains using optical imaging methods and digital image processing technology (Duan et al., 2011a; Duan et al., 2011b; Huang et al., 2013; Huang et al., 2022). Some works combine scanning tiled rice grains with image processing techniques to analyze rice grains’ morphological and color traits (Whan et al., 2014; Wu et al., 2019). An automated analysis software for rice panicle traits based on traditional digital image processing methods has been developed, which can estimate the number of grains on rice panicles with high throughput (Al-Tam et al., 2013). However, those methods require specialized threshing, transmission, and imaging environment, resulting in high image acquisition costs. The threshing process is prone to damage the rice and affect the accuracy of the final results.

In contrast, methods that do not require threshing have higher efficiency and stronger generalization ability. Many studies directly attempt to extract grains and related traits from rice panicle images. The two-dimensional image information of rice panicles can also be used for modeling, a correction-model-referred on-panicle grain counting method was proposed based on the area of the rice panicle and its edge contour wavelet analysis and achieves an average accuracy of 94% compared to the results of manual counting (Gong et al., 2018). The area of the panicle was also used to directly predict yield (Zhao et al., 2019). Deep learning technology has also been widely used in rice counting, positioning, and segmentation (Wu et al., 2019; Deng et al., 2022). Due to the frequent adhesion and occurrence of natural shielding on rice panicles, methods that do not require threshing can usually only obtain quantitative traits of rice but cannot obtain morphological traits. Some researchers have reduced shielding by separating several branches of a single rice panicle and extracting quantitative and morphological traits of rice grains (Gong et al., 2018; Wang et al., 2022). However, separating rice branches is time-consuming and fragile, and it is still impossible to avoid the impact of rice adhesion on the results. Some advanced imaging systems, such as the X-ray imaging system, have also been used to extract rice traits, but high costs and low efficiency limit the promotion of such methods (Su and Chen, 2019; Hu et al., 2020; Yu et al., 2021). Therefore, developing a low-cost phenotyping method for analyzing comprehensive on-panicle rice grain traits with high throughput, high accuracy, and without complex pretreatment is necessary.

This study proposed a high-throughput phenotyping method for extracting on-panicle rice grain traits without grain threshing and branch separating. Color images of individual rice panicles are efficiently obtained based on visible light scanning imaging technology. The cascaded Faster R-CNN model and an improved Pix2Pix model were used to detect, segment, and restore every on-panicle rice grain. Based on the processing results, fifteen rice grain traits are automatically calculated in the designed image processing pipeline.

## 2 Materials and methods

### 2.1 Collection of rice panicle

This study randomly selected rice panicles from eight varieties, including three japonica and five indica varieties. Six were planted in Wuhan, Hubei province (30.27°N,114.2°E) and harvested in mid-July 2021. The other two were planted in Sanya, Hainan Province (18.24°E,109.50°E) and harvested in early July 2022. Whether in Wuhan or Sanya, all materials were planted in the same experimental field, using different plots to distinguish different varieties. In terms of field management, both experimental fields followed the conventional field method of maintaining a certain water level throughout the entire growth period. The conditions were strictly identically controlled, except for differences in varieties. Field maintenance, including weeding and pest control, was performed by professionals throughout the growth period. Twenty panicle samples from each variety were randomly selected for imaging and analysis. Specific information for each variety is shown in **Table 1**.

**Table 1:** Variety information.

Rice variety	Subspecies	Growing area	Harvested time
Jiujiuxinxiang	indica	Sanya	2022.07
Hongxiangyou3	japonica	Sanya	2022.07
Kendao1867	japonica	Wuhan	2021.07
Kenyan1803	japonica	Wuhan	2021.07
Wangdao2	indica	Wuhan	2021.07
Wendao21	indica	Wuhan	2021.07
Z98-308	indica	Wuhan	2021.07
Ganzhi	indica	Wuhan	2021.07

### 2.2 Visible light scanning imaging method

**Figure 1:**
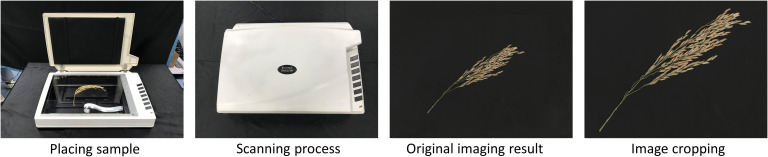
Visible light scanning imaging process.

All images in the experiment were collected by a visible light scanner (Uniscan M1, Tsinghua Unigroup, China). The rice panicles were placed on the scanning panel. A computer with a 64-bit Windows operating system was connected to set the scanning parameters and control the scanner. The scanning mode is a charge-coupled device (CCD). The scanning resolution was 600 dpi, and the image size was 7200 * 10200 pixels. Then crop the image to 5700 * 6800 pixels to remove some background area (**Figure 1**). According to scanner parameters, each pixel corresponds to an actual size of 0.0423 mm. Under this parameter, the time for single imaging and storing the result is approximately 60 seconds.

### 2.3 On-panicle rice grain traits extracting algorithm based on deep learning

**Figure 2:**
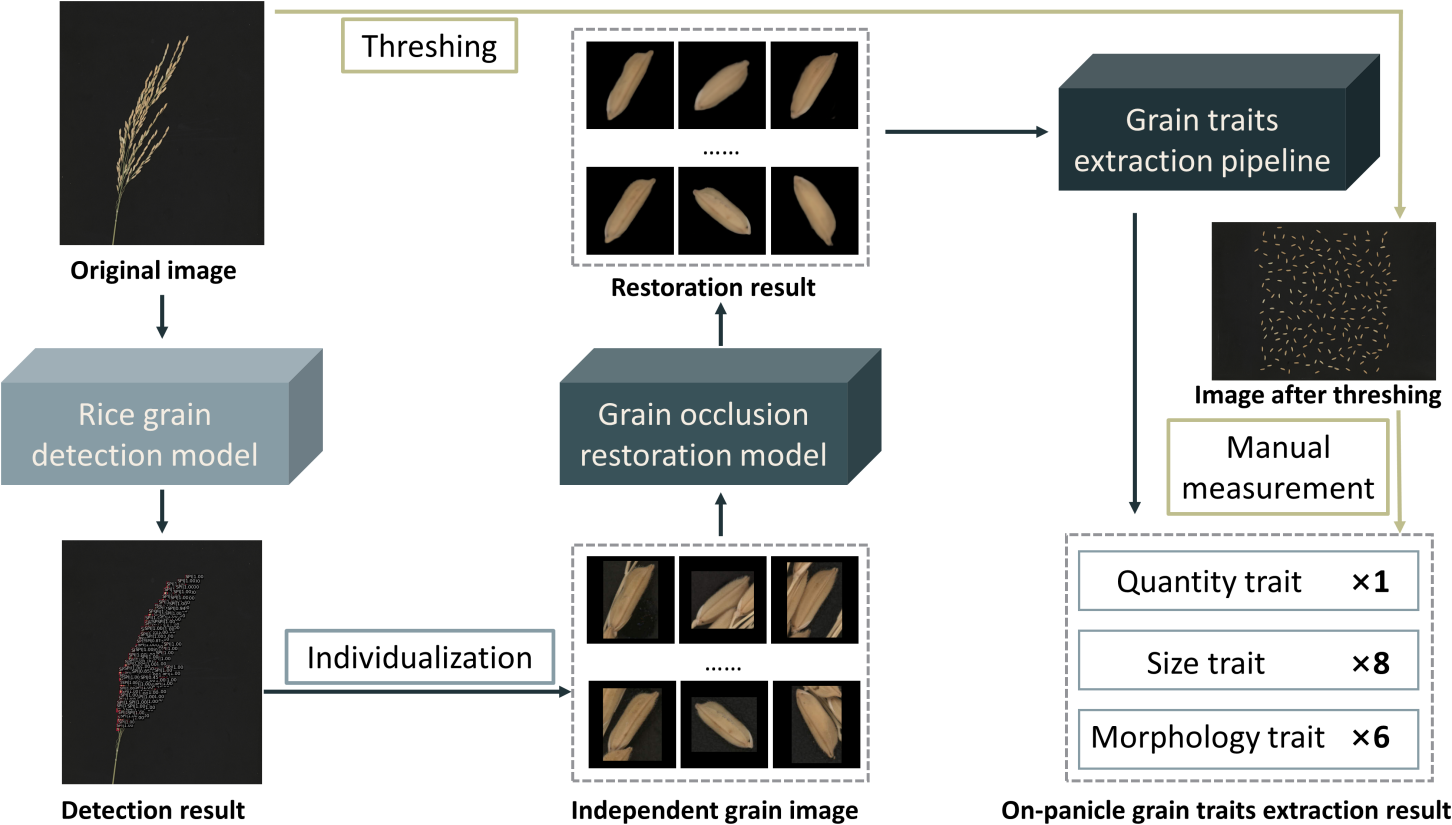
Workflow of on-panicle rice grain traits extracting algorithm.

The on-panicle rice grain extracting algorithm based on visible light scanning imaging results comprises three cascade modules (**Figure 2**). Firstly, the rice grain detection model detects each grain on the panicle and outputs a region of interest (ROI) local image of the target area. Secondly, the grain occlusion restoration model is used to restore each output result in the upper part to compensate for possible information loss caused by occlusion. Thirdly, the grain trait extraction pipeline is used to calculate rice grain-related phenotyping traits, including one quantitative trait, eight size traits, and six morphological traits.

#### 2.3.1 Rice grain detection model based on Faster R-CNN

Faster R-CNN (Ren et al., 2015) is a convolutional neural network model for target detection tasks proposed by Ren Shaoqing and He Kaiming based on R-CNN (Girshick et al., 2014) and Fast R-CNN (Girshick, 2015). This network skillfully solves the problem of slow training and prediction speed for R-CNN and Fast R-CNN by simultaneously training classification and regression tasks. It proposes a Regional Proposal Network (RPN), which enables the network to conduct end-to-end training. Since its introduction, Faster R-CNN has attracted the attention of many researchers and has been successfully applied in many fields.

**Figure 3:**
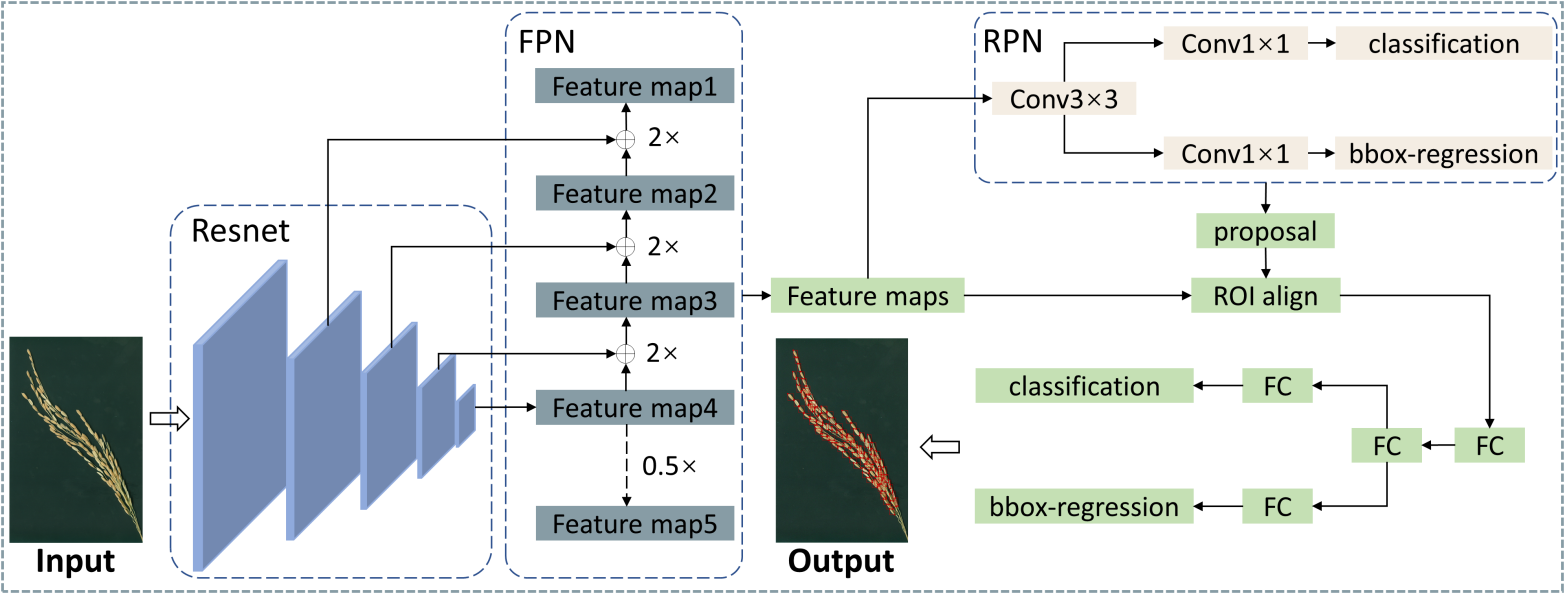
Designed rice grain detection model based on Faster R-CNN.

The Faster R-CNN mainly includes four parts: feature extraction network, region proposal network (RPN), ROI Pooling module, and classification/regression module. This architecture has good performance in general target detection tasks. Still, in this study, the research targets are small and densely distributed, making it difficult for the original Faster R-CNN network structure to detect grains accurately. Feature pyramid networks (FPN) significantly improve the detection effect of models for small targets by fusing feature maps of different depths (Lin et al., 2017). Therefore, to improve the model’s accuracy for detecting grains in the ear, this study incorporated the FPN module into the Faster R-CNN. The overall structure of the designed rice grain detection model is shown in **Figure 3**.

#### 2.3.2 Rice grain occlusion restoration model based on improved Pix2Pix

**Figure 4:**
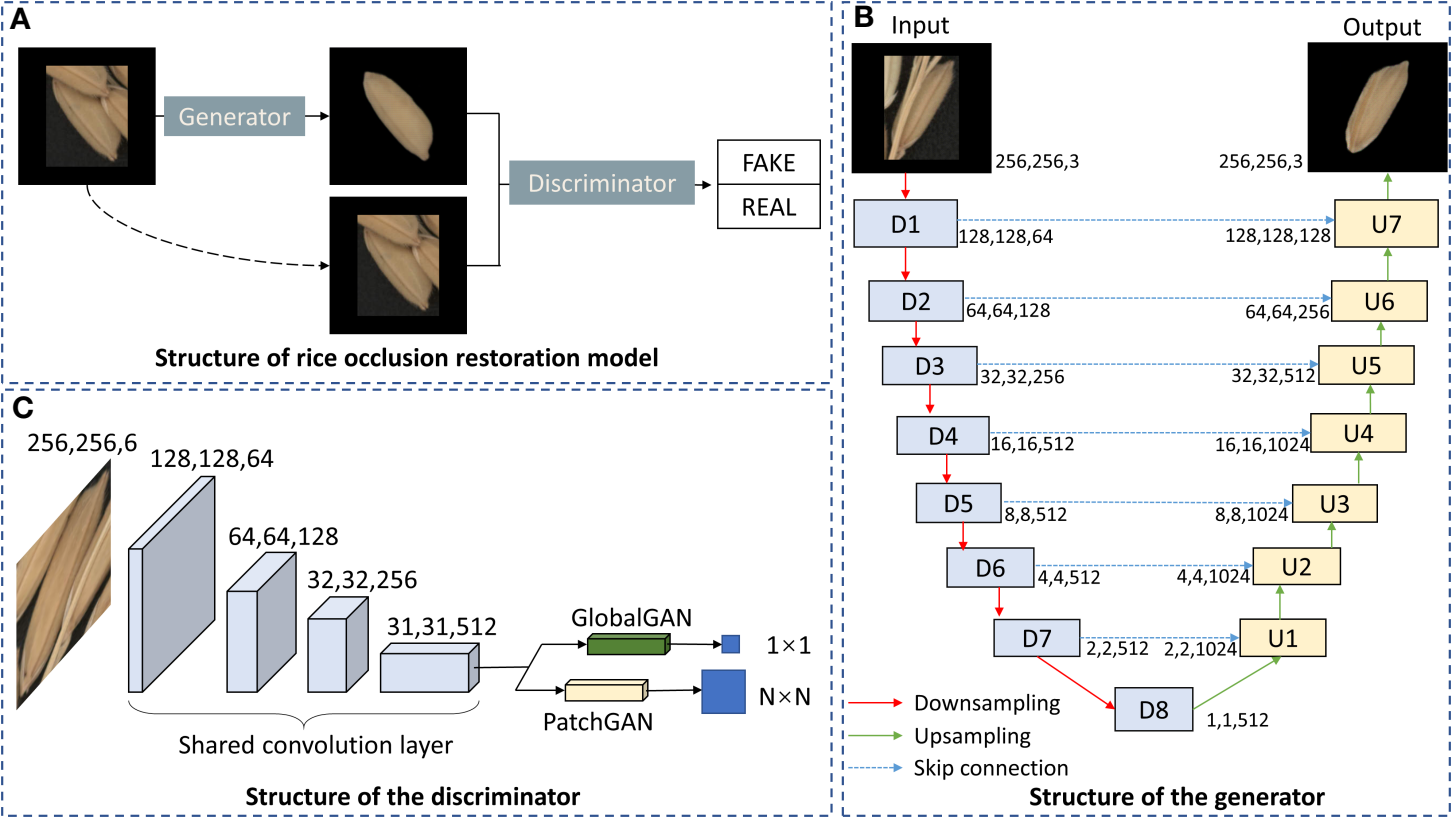
Structure of rice occlusion restoration model based on improved Pix2Pix. **(A)** Structure of rice occlusion restoration model. **(B)** Structure of the generator. **(C)** Structure of the discriminator.

Pix2Pix (Isola et al., 2017) is an image translation model based on a conditional generative adversarial network (CGAN) (Mirza and Osindero, 2014). Pix2Pix learns a mapping between the input and output images by conditioning the input image to obtain the specified output image. The U-Net structure will be adopted as the generator in Pix2Pix (**Figure 4B**). Different from the traditional encoder-decoder structure, U-Net (Ronneberger et al., 2015) uses skip connections between corresponding encoder and decoder layers to preserve low-level features that may be lost during downsampling. These skip connections concatenate the feature maps from the encoder with those of the corresponding decoder layer, significantly improving image details’ reconstruction. The key for Pix2Pix training is the discriminator, which is named PathchGAN. Unlike traditional GlobalGAN discriminator, the output of PatchGAN is not a scalar but an N×N two-dimensional matrix, and each element of this matrix corresponds to a patch in the original image. By discriminating each patch, PatchGAN can provide better feedback to the generator about the local consistency of the generated images.

In the process of grain restoration, the restoration effect of global and local image details will affect the extraction of traits. In order to obtain more accurate grain traits, we need to comprehensively consider the global and local details of the restored grain image. Therefore, GlobalGAN and PatchGAN are fused as the discriminator of the grain occlusion restoration model (**Figure 4C**).

In summary, the complete grain occlusion restoration model (**Figure 4A**) mainly includes the following three parts: the generator of the U-Net structure, the discriminator of the fusion of GlobalGAN and PatchGAN, and the CGAN architecture to train the network. The overall structure of the grain occlusion restoration model is shown in **Figure 4**.

#### 2.3.3 Image-based automatic extraction pipeline for rice grain traits

**Figure 5:**
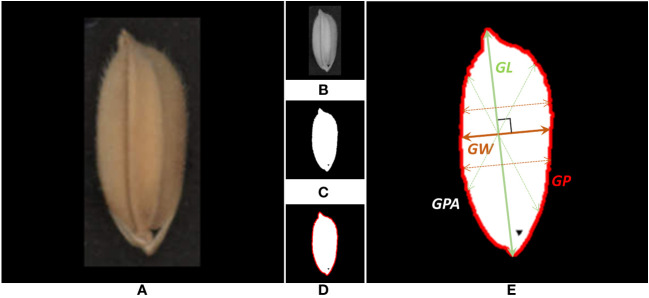
Automatic extraction pipeline for rice grain trait. **(A)** Single-grain scanning image. **(B)** Grayscale of the red channel. **(C)** Binary image after OTSU segmentation. **(D)** Outside contour of grain. **(E)** Morphological traits of grain.

An automatic extraction pipeline for grain traits was designed to process each rice grain image obtained in the previous step. The automatic extraction pipeline, as shown in **Figure 5**, was developed with Python language and OpenCV (Bradski, 2000), an open-source image processing toolkit. Firstly, the RGB image of each independent rice grain (**Figure 5A**) was used as input to the pipeline. Secondly, the red channel (**Figure 5B**) was extracted from the RGB image of the grain. Compared with other channels, the contrast of the red channel was more obvious, which could better separate the grain from the background. Thirdly, the OTSU algorithm was used to automatically generate the optimal segmentation threshold and binarize the gray-scale image (**Figure 5C**). Fourthly, extracting the outer contour (**Figure 5D**) based on binary images was the basis for further trait calculation. Fifthly, the projection area, perimeter, and length of the rice grain are obtained by measuring the area of the grain, the outer contour length, and the distance between the farthest two points on the contour (**Figure 5E**). Obtain the intersection point of lines perpendicular to the major axis and the contour and use the maximum value of the distance as the grain width. Grain length/width ratio, perimeter/area ratio, equivalent ellipse and circularity could be further calculated by the previous traits. **Table 2** shows the total fifteen on-panicle rice grain-related traits that can be extracted.

**Table 2:** On-panicle rice grain phenotyping traits evaluated in this study.

Type	Traits	Abbreviation	Mean ± SD	Unit
Quantity trait	Grain number	GN	109.450 ± 48.782	–
Size trait	Mean value of grain length	MGL	8.607 ± 0.697	mm
	Standard deviation of grain length	SGL	0.589 ± 0.156	mm
	Mean value of grain width	MGW	2.770 ± 0.139	mm
	Standard deviation of grain width	SGW	0.248 ± 0.052	mm
	Mean value of grain projection area	MGPA	18.140 ± 1.307	mm^2^
	Standard deviation of grain projection area	SGPA	1.875 ± 0.332	mm^2^
	Mean value of grain perimeter	MGP	21.313 ± 1.481	mm
	Standard deviation of grain perimeter	SGP	1.386 ± 0.412	mm
Morphology trait	Mean value of grain area/perimeter ratio	MGAPR	0.851 ± 0.037	–
	Standard deviation of grain area/perimeter ratio	SGAPR	0.070 ± 0.011	–
	Mean value of grain circularity	MGC	0.507 ± 0.048	–
	Standard deviation of grain circularity	SGC	0.048 ± 0.007	–
	Mean value of grain length/width ratio	MGLWR	3.148 ± 0.340	–
	Standard deviation of grain length/width ratio	SGLWR	0.377 ± 0.100	–

Symbol "-" indicates that the parameter has no units.

## 3 Result

### 3.1 Accuracy evaluation of on-panicle rice grain detection model

This experiment was run on a Dell Precision3650 server with Intel core i7-11700k CPU (32 GB memory) and NVIDIA GeForce RTX 3090 GPU (24 GB graphic memory). The software environment for deep-learning model training uses Python language under an Ubuntu operation system with Pytorch deep-learning framework.

Since the selection of model parameters will directly affect the final performance of the model, all the relevant parameters were adjusted before the training of the Faster R-CNN model for the situation where the number of on-panicle rice grains panicle is large, the size is small, and there is a certain degree of mutual occlusion to be detected in this study. The main parameters are shown in **Table 3**.

**Table 3:** Main hyperparameter settings of rice grain detection model and occlusion restoration model.

Model	Hyperparameter	Setting
Rice grain detection model based on Faster R-CNN	Img_size	1425 x 1700 pixels
	NMS threshold	0.74
	Batch size	1
	Optimizer	SGD
	Learning rate	0.02
	Momentum	0.9
	Weight decay	0.0001
	RPN proposal number	4000
	Maximum epoch number	100
Rice grain occlusion restoration model based on improved Pix2Pix	Img_size	256x256 pixels
	Batch size	64
	Optimizer	Adam
	Learning rate	0.0002
	Maximum epoch number	500

This study obtained 160 images of rice panicles in natural form. After manual labeling, all the images were divided into training, verification, and test sets in a 2:1:1 ratio. Based on the number of grains detected by the grain detection model and the actual number of grains in the panicle, the R^2^ coefficient, mean absolute percentage error (MAPE) and root mean square error (RMSE) were used to measure the accuracy of the grain detection model. Mean average precision (mAP) was used to evaluate the accuracy of on-panicle rice grain location.

The R^2^, MAPE and RMSE were calculated by the following equation:


(1) $$\begin{array}{c} {\text{R}}^{2}=1-\frac{\sum_{i=1}^{n}{\left({\hat{y}}_{i}-y_{i}\right)}^{2}}{\sum_{i=1}^{n}{\left(y_{i}-\bar{y}\right)}^{2}} \end{array}$$

(2) $$\begin{array}{c} \text{MAPE}=\frac{1}{n}\sum_{i=1}^{n}\frac{\left|{\hat{y}}_{i}-y_{i}\right|}{y_{i}}\times 100\% \end{array}$$

(3) $$\begin{array}{c} \text{RMSE}=\sqrt{\frac{1}{n}\sum_{i=1}^{n}{\left({\hat{y}}_{i}-y_{i}\right)}^{2}} \end{array}$$

${\hat{y}}_{i}$ is the trait parameters extracted from the grain image after restoration,

$y_{i}$ is the true trait parameter,

  $\bar{y}$ represents the average value of grain trait in samples

**Figure 6:**
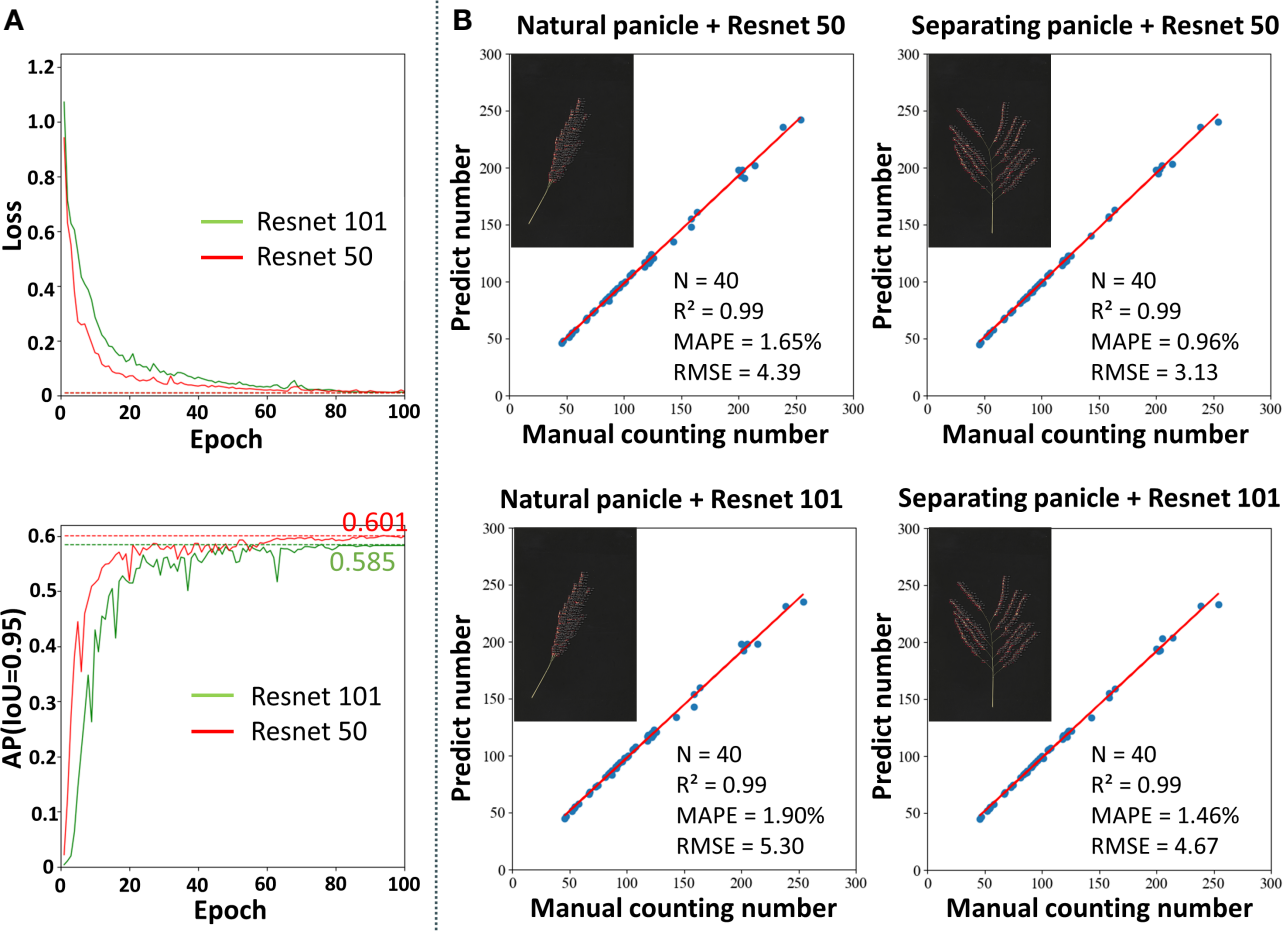
Performance of the rice grain detection model. **(A)** The training loss and AP curves with Resnet50 and Resnet101. **(B)** Comparison between the number of grains prediction by the model and manual counting.

The two most commonly used feature extraction networks (He et al., 2016), based on Resnet50 and Resnet101, were used for performance comparison to select the network depth appropriate for rice grain detection (**Figure 6A**). At the beginning of training, the losses of both models decreased rapidly. After training for 20 epochs, the speed of loss reduction slowed down and converged after 80 epochs, and the model achieved the optimal state. Faster R-CNN using Resnet50 as a feature extraction network showed faster and better convergence. **Figure 6A** shows the AP variation curve of the Faster R-CNN model on the validation set. The changing trend of AP was opposite to the changing trend of loss. Finally, the Faster R-CNN model using Resnet50 as a feature extraction network achieved a higher AP, proving that Resnet50 was more suitable for detecting grains in the panicle than Resnet101. In addition, it was found that the AP reached 0.965, 0.933 and 0.601 when IoU was 0.50, 0.75 and 0.95, respectively.

The Faster R-CNN model marks the detected grains on the original map as detection frames, so the number of detection frames on the resulting map is the number of detected grains. Two types of image data, including natural morphology and separating the branch, were used to verify the accuracy of the network. The counting results are shown in **Figure 6B**. In all cases, the Faster R-CNN model can accurately count the on-panicle rice grains, with R^2^ reaching 0.99. In addition, the counting accuracy of the Faster R-CNN model using Resnet50 as a feature extraction network was slightly higher than that of Resnet101, both in natural morphology and after artificially separating branches of the panicle. Compared with the case of separate branches, the model showed a slight decrease in accuracy in the case of the natural panicle. Specifically, the MAPE value increased by 0.69% and the RMSE value increased by 1.26. In conclusion, for natural rice panicles, the on-panicle rice grain detection model proposed in this study achieved 1.65% on MAPE and 4.39 on RMSE.

### 3.2 Performance evaluation of on-panicle rice grain occlusion restoration model

The dataset of the on-panicle rice grain occlusion restoration model must be paired. Various situations that may occur under natural conditions can be simulated by manually adjusting the grain occlusion ratio. Finally, 2000 pairs of images, set at an 8:2 ratio, were used in the model’s training set and verification. The hyperparameter settings for the model are shown in **Table 3**.

**Figure 7:**
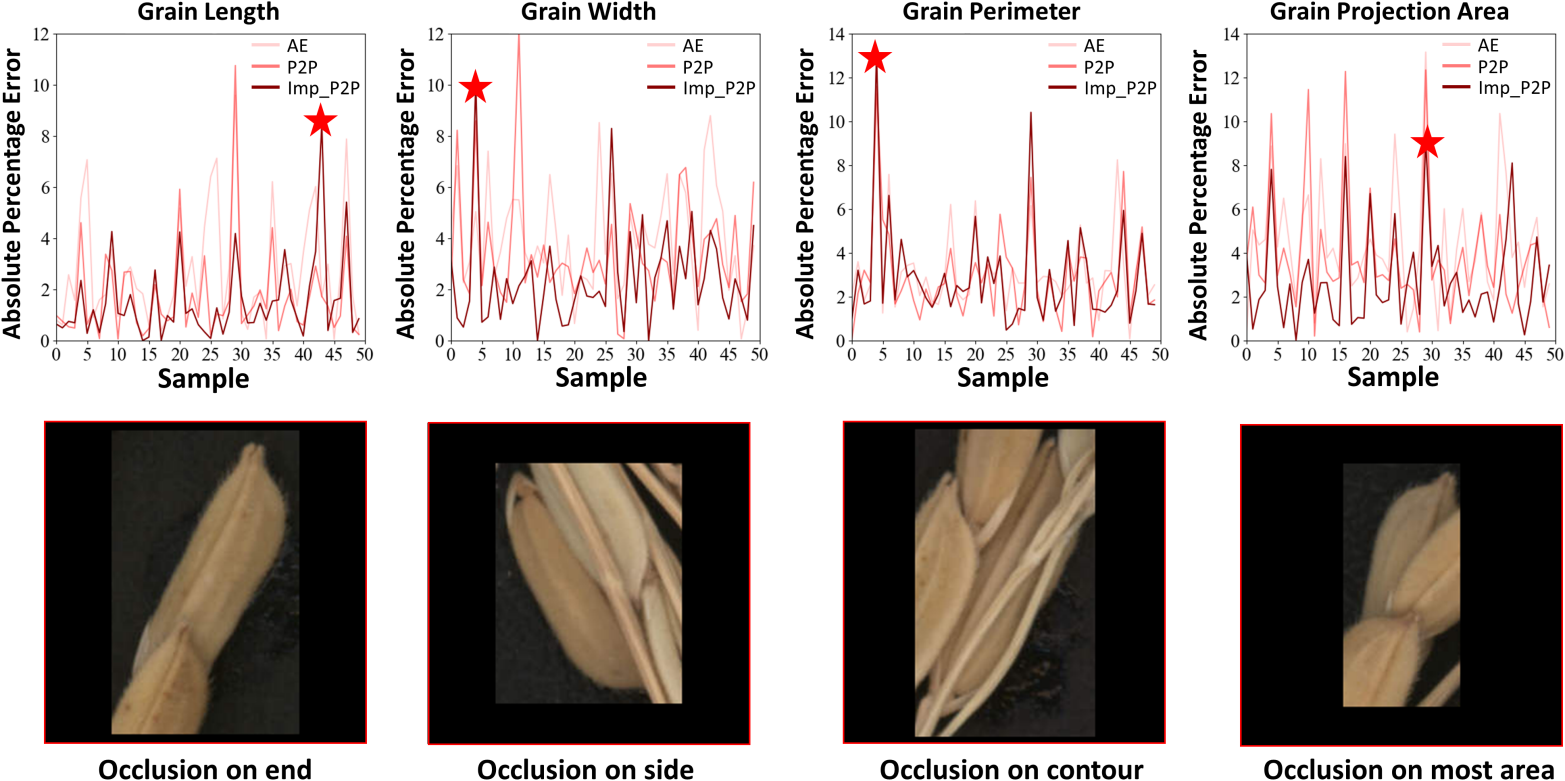
The restoration performance of each model in grain traits.

**Figure 8:**
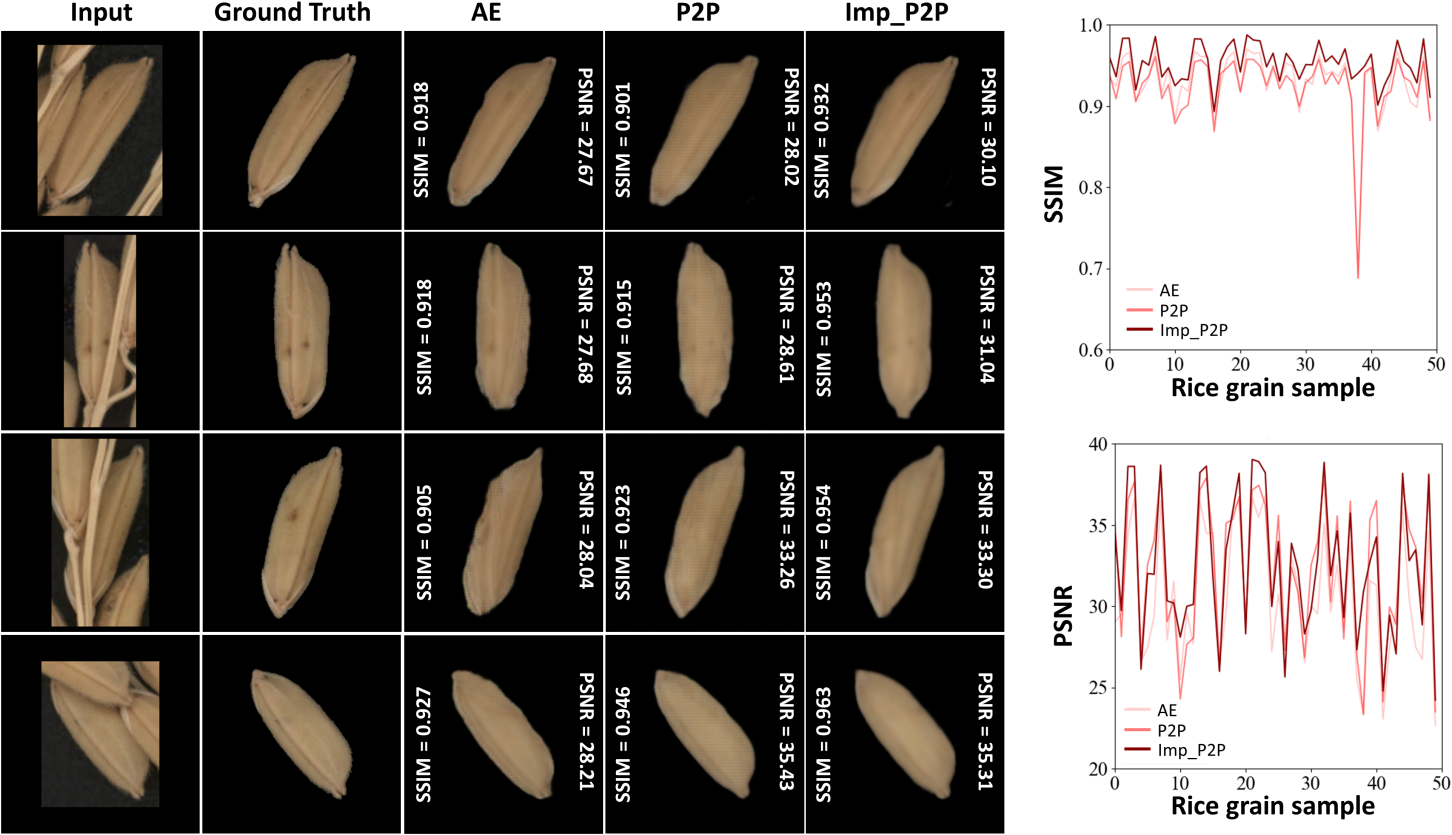
The restoration performance of each model in SSIM and PSNR.

Fifty pairs of images with varying degrees of occlusion were used to test the performance of the restoration model. The MAPE value was used to verify the model’s restoration performance from the perspective of trait calculation (**Figure 7**). Peak signal-to-noise ratio (PSNR) and structural similarity (SSIM) were used to evaluate the model’s ability in image feature restoration (**Figure 8**). To prove the excellent performance of the proposed model (Improved Pix2Pix, ImpP2P), this paper used the same hyperparameters to train an auto-encoder-based generating model (AE) and the original Pix2Pix model (P2P).

PSNR and SSIM have been commonly used evaluation metrics in image restoration. PSNR is based on the error of corresponding pixels with the dB unit. The higher the PSNR, the smaller the image distortion. SSIM is a full-reference image quality evaluation metric that measures the similarity of images from three aspects: luminance, contrast, and structure. Its value range is from 0 to 1. The larger the SSIM, the smaller the image distortion. Their calculation formulas are shown in equations 4 and 5, respectively.


(4) $$\begin{array}{c} PSNR=10*\log_{10}\left(\frac{MAX_{I}^{2}}{\frac{1}{mn}\sum_{i=0}^{m-1}\sum_{j=0}^{n-1}X\left(i,j\right)-Y{\left(i,j\right)}^{2}}\right) \end{array}$$

Where X(i,j) and Y(i,j) represent the pixel values of the real grain image and the grain image after restoration at the coordinate (i,j) . m and *n* represent the height and width of the image. In this paper, both m and n are 256. $MAX_{I}$ represents the maximum possible pixel value for the image, which is 255 in the case of an 8-bit binary grayscale image.


(5) $$\begin{array}{c} SSIM\left(x,y\right)=\frac{\left(2\mu_{x}\mu_{y}+c_{1}\right)\left(2\sigma_{xy}+c_{2}\right)}{\left(\mu_{x}^{2}+\mu_{y}^{2}+c_{1}\right)\left(\sigma_{x}^{2}+\sigma_{y}^{2}+c_{2}\right)} \end{array}$$

Where *x* and *y* represent the unoccluded grain image and the grain image after restoration, respectively. $\mu_{x}$ and $\mu_{y}$ represent the mean value of image respectively, and $\sigma_{x}$ and $\sigma_{y}$ represent the variance of image, respectively. $\sigma_{xy}$ represents the covariance of the images. $c_{1}$ and $c_{2}$ are constants to avoid division by zero.

As shown in **Figure 7**, the improved Pix2Pix model performs best on all four grain traits. Regarding grain length, the MAPE of AE and P2P is 2.76% and 1.68%. As for the improved Pix2Pix model, it achieves 1.50%. They are 4.01%,3.48% and 2.41% in grain width for AE, P2P and ImpP2P. In grain perimeter, they are 3.21%, 2.92% and 2.85% for AE, P2P and ImpP2P. In grain projection area, they are 4.42%, 3.84% and 2.74% for AE, P2P and ImpP2P. For individual rice grains, when occlusion occurs on the end, side, surround, and large area of the rice grain, it will cause significant errors in the length, width, perimeter, and area measurement, respectively.

**Figure 9:**
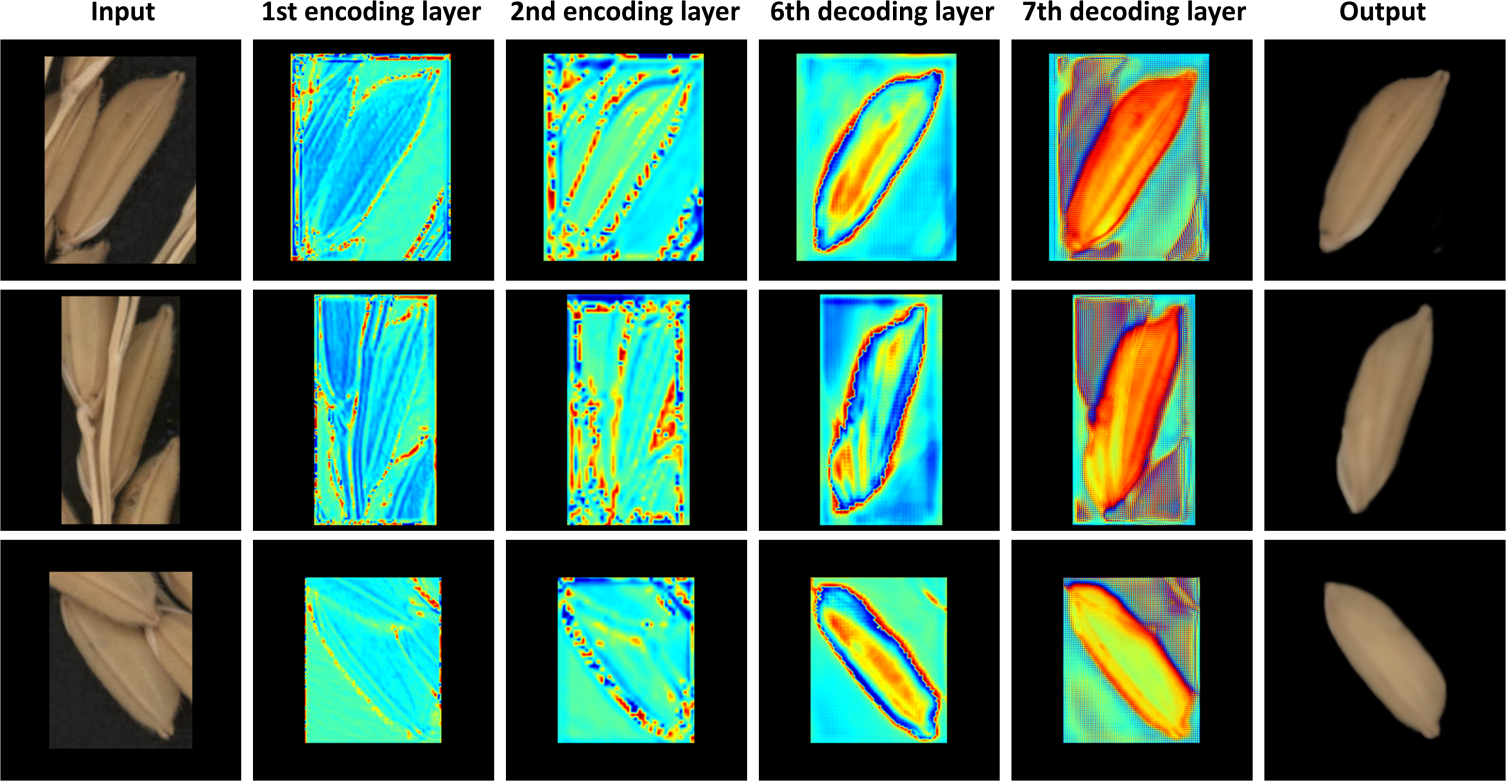
The visualization of typical convolutional layers in occlusion restoration model.

**Figure 8** shows four representative occlusion situations. All models can restore the approximate shape of the grain, and the ImpP2P model performs best in terms of overall structure and signal-to-noise ratio. For 50 rice grains with various degrees of occlusion, the average SSIM of ImpP2P achieves 0.953, better than AE (0.929) and P2P (0.924). The average PSNR of ImpP2P, AE and P2P are 32.49, 30.59 and 32.22. The visualization of four typical convolutional layers is shown in **Figure 9**, they are feature maps of the 1st encoding layer, the 2nd encoding layer, the 6th decoding layer and the 7th decoding layer. It can be found that the network has learned some features in different abstractive layers. In the encoding layers, the edge information of rice grains and significant internal areas are more concerned by the network. Correspondingly, in the decoding layers, the edge and internal center regions of the target are first restored and eventually extended to the entire rice grain.

### 3.3 Reliability verification of on-panicle rice grain phenotyping traits

**Figure 10:**
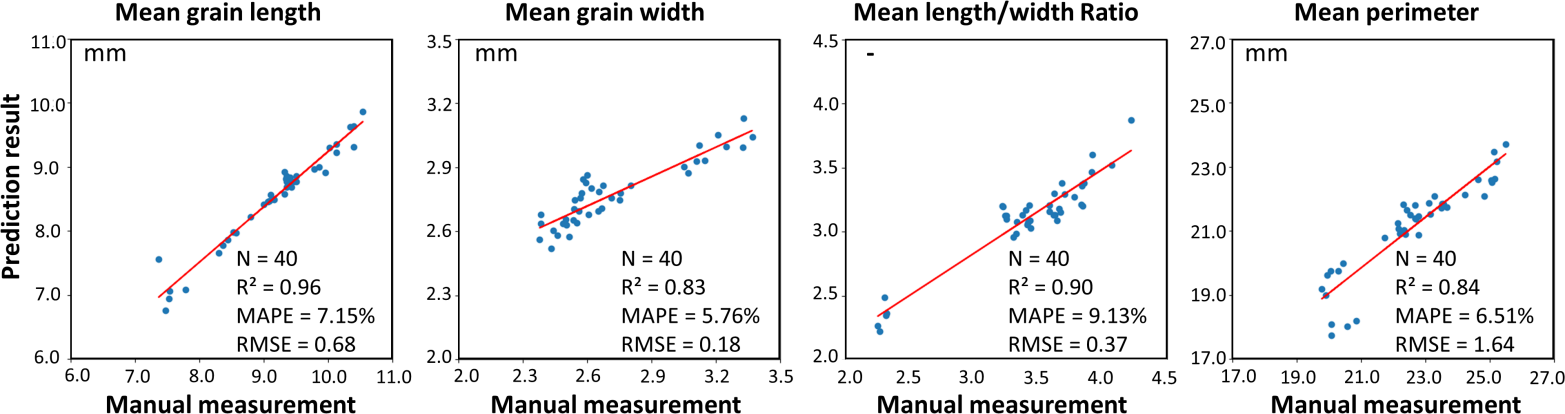
Analysis of extraction results of four morphological traits.

Forty rice panicles selected randomly from eight varieties were used as samples to verify the reliability and robustness of the proposed on-panicle rice grain phenotyping method. The grain number of all rice panicles is distributed between 45 and 250. The result of the fifteen traits is shown in **Table 2**, and the average time-consuming for the processing and trait calculation of each rice panicle image is about 10 seconds. The time-consuming will inevitably increase with the growth of on-panicle grain numbers. Four morphological traits directly related to rice quality, mean grain length, mean grain width, mean grain length-width ratio and mean grain perimeter were used to compare the result by the method with the ground truth, which was obtained by manually measuring rice grains after threshing (**Figure 2**). For all grains on each panicle, mean length, mean width, mean length/width ratio and mean perimeter extracted by the method proposed in this paper are compared with the results of manual extraction (**Figure 10**), and the R^2^ values between them reach 0.96, 0.83, 0.90 and 0.84. The MAPE values of the method versus the manual measurement for the four traits are 7.15%, 5.76%, 9.13% and 6.51%. Their RMSE values are 0.68mm, 0.18mm, 0.37 and 1.64mm, respectively. According to the conclusion in **Figure 6**, the R^2^, MAPE, and RMSE values for grain number counting are 0.99, 1.65% and 4.39.

**Figure 11:**
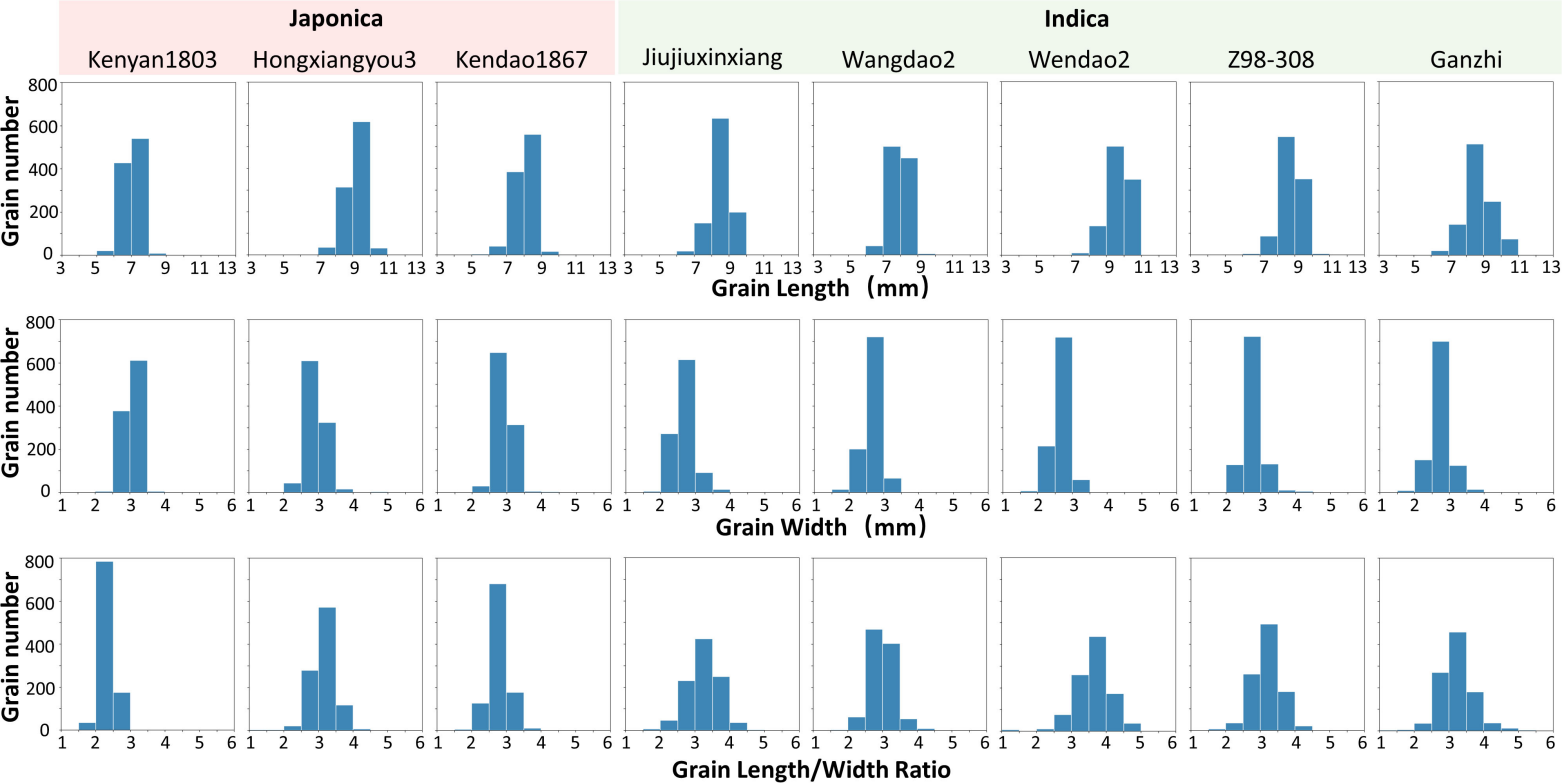
Analysis of on-panicle grain traits of eight rice varieties.

Referring to the concept of thousand-grain weight commonly used in rice seed evaluation, the thousand-grain length, thousand-grain width, and thousand-grain length/width ratio of eight varieties were obtained. The histograms of the distribution quantities on three traits of the samples are shown in **Figure 11**. Overall, most rice grains are 5-11mm long for all varieties, the grain width of samples from subspecies japonica is larger, appearing thicker and shorter compared to subspecies indica, while samples from subspecies indica are slender. As shown in **Figure 11**, rice grains of Kenyan1803 are generally short, most of them in the range of 6-8mm. Grains of Ganzi have a prominent length; most are larger than 8mm, and a considerable part is higher than 10mm. As for grain width, most of the grain widths of all varieties are between 2 and 4mm, and some varieties, such as Wangdao2 and Ganzhi, have very few grains with a grain width of less than 2mm. For Kenyan1803, almost all the grains are wider than 2.5mm. The length/width ratio is one of rice grains’ most crucial reference traits, and the eight varieties are mostly distributed between 2 and 5. The grain distribution range of Kenyan1803 is the most concentrated, and most are between 2 and 3, indicating that the grains of this variety have little difference in shape. On the contrary, the grain length-width ratio distribution of Jiujiuxinxiang, Wendao21, Z98-308 and Ganzhi is relatively dispersed, indicating that the grains of these varieties have significant differences in shape.

## 4 Discussion

The number of grains per panicle and grain morphological-related traits of rice varieties are essential reference data for rice breeding and functional identification of crucial genes. High throughput, convenient, and economical phenotypic trait evaluation methods are crucial. In previous work, the accuracy and efficiency of measuring grain-related traits were often contradictory. High-accuracy methods often rely on complex mechanical equipment and post-processing algorithms. In contrast, simple and efficient imaging and processing methods are challenging to obtain accurate and comprehensive phenotyping traits. These problems limit the promotion and development of these phenotyping methods. This study proposed a method based on visible light scanning imaging and deep learning technology for on-panicle rice grain traits, which balanced measurement efficiency and accuracy. Visible light scanning equipment is inexpensive (The price is less than one percent or even lower than that of large seed testing equipment and X-ray imaging equipment), readily available, and can provide stable imaging results in laboratory and field environments. The method in this study does not require complex sorting and threshing of samples, which often takes one minute or even longer for a single rice panicle. The method in this study could potentially expand to the detection of rice panicle and grain traits at multiple growth stages of rice.

Due to the natural occlusion of on-panicle rice grains, previous measurement methods can only estimate the number of rice grains and other morphological traits after separating the branches. Separating and fixing branches is time-consuming and fragile, and the accuracy of trait extraction is easily affected by the degree of separation. Even so, avoiding the possible occlusion between adjacent grains is impossible. Deep learning technology provides a way to solve this problem. This study proposed a cascade model based on the Faster R-CNN model and improved the Pix2Pix model to achieve accurate counting and occlusion restoration of the on-panicle grains. From the restoration results, morphological traits of grains can be extracted without panicle separation. For rice panicles with grain numbers between 45 and 250, the grain detection will be completed in about 1 second, and the extraction of traits will take about 10 seconds. The sufficient experimental result proves the high accuracy and reliability of the method.

The method proposed in this study achieves high-throughput and high-accuracy extraction of on-panicle rice grain traits without separating the branch. However, there are still some directions for improvement. Firstly, portable visible-light scanning devices could be developed for researchers to use in field environments. Secondly, simultaneous imaging of multiple rice panicles is possible, which can double the efficiency of trait analysis. For the deep learning model, more occlusion scenes and occlusion degrees can be designed to improve the model’s accuracy when applied to multiple varieties.

## 5 Conclusions

This study proposed a high-throughput and separating-free method for extracting on-panicle rice grains phenotyping traits based on visible light scanning imaging and deep learning. Samples from eight varieties were used to verify the accuracy of the method. The results showed that the method proposed in this paper could obtain images of rice panicles within 60 seconds and automatically extract 15 traits of on-panicle grains in about 10 seconds. Compared with manual measurement, the R^2^ values of the method on grain counting, grain length, grain width, grain length/width ratio and grain perimeter reach 0.99, 0.96, 0.83, 0.90 and 0.84, respectively. The difference in the distribution of grain traits among different varieties indicates that this method can effectively distinguish varieties and help screen high-quality traits. In general, the method proposed in this paper can be used to realize the rapid measurement of rice grain traits and has the potential to be extended to the field environment and other crops.

## Data availability statement

The datasets presented in this study can be found in online repositories. The names of the repository/repositories and accession number(s) can be found below: https://github.com/BME-PhenoTeam/Method-for-on-panicle-rice-grain-detection.

## Author contributions

YL and LY designed the research. YL and JW performed the experiments, analyzed the data, and wrote the manuscript. LF, LY, and QL helped to perform the experiments. QL and LY supervised the project. All authors contributed to the article and approved the submitted version.
